# Professional Self-Esteem of Turkish Midwifery Students: A Mixed-Methods Approach

**DOI:** 10.3390/healthcare11091214

**Published:** 2023-04-25

**Authors:** Zehra Baykal Akmeşe, Ummahan Yücel

**Affiliations:** Department of Midwifery, Faculty of Health Science, Ege University, Izmir 35575, Turkey; zehra.baykal@ege.edu.tr

**Keywords:** professional self-esteem, midwifery, student

## Abstract

Professional self-esteem (PSE) is the individual’s judgment of worthiness formed regarding the self-attributions related to the individual’s profession. A well-developed PSE is important for midwives to be successful in their profession, have a strong sense of belonging, and see the profession as important and valuable. This study aims to determine the perception of PSE among midwifery students, explain how their perceptions are formed, and reveal how they perceive the midwifery profession and how the components that make up this perception determine their PSE. Students’ average PSE score was analyzed, and the study was conducted using an explanatory sequential, which is a mixed-method approach, to interpret the results of the questionnaire and comparative analysis in more detail through direct interviews with university midwifery program students in western Turkey. Quantitative data were collected using the Individual Data Collection Form and the Professional Self-Esteem Scale. One-way analysis variance and independent sample *t*-tests were used to analyze the quantitative data. The students with the highest and lowest scale scores from each class level were interviewed using a Semi-Structured Interview Form. Thematic analysis was applied to analyze the qualitative data. The mean PSES score was 117.51 ± 17.60. The *t*-test analysis result shows that there was a significant difference between the PSES score and choosing the midwifery department willingly, believing that they could perform the midwifery profession before and after starting the midwifery education (*p* < 0.05). Three main themes were obtained: the meaning of being a midwife, deciding to become a midwife, and the difficulties of being a midwife. It can be said that the main force behind the students’ belief that the difficulties of the midwifery profession can be overcome is their strong love for the midwifery profession and their internalization of its importance.

## 1. Background

Professional self-esteem (PSE) is defined as ‘an individual’s judgment of worthiness formed regarding the self-attributions related to the individual’s profession’ [1]. As well as the meaning and importance that the individual attributes to his profession, PSE also means that he gets satisfaction from and is compatible with that profession’ [2]. Midwifery is a profession with increasing significance throughout the world [3]. Midwives have several roles and responsibilities within the scope of the first, third, and fifth Sustainable Development Goals of the World Health Organization [4]. Midwives play an active role in combating poverty. Its effects on women’s health include reducing maternal and neonatal mortality rates, ending preventable deaths in children under the age of five, ensuring access to qualified reproductive health services, and promoting gender equality. In addition, within the scope of these goals, midwives directly contribute to social development in the areas of eliminating violence against women and promoting the active participation of women in political, economic, and public life [4]. In addition to deep scientific knowledge about every stage of the holistic protection and development of women’s health, midwives must have high PSE to achieve these ambitious goals. Midwifery is not just using the knowledge and skills, but also reflecting the high PSE in the professional experience [3,4,5]. A well-developed PSE is critical for midwives to be successful in their profession, have a strong sense of belonging, and see the profession as important and valuable. The knowledge, skills, and proficiency acquired during the education of midwifery students who will be the midwives of the future should be transferred to practice with a well-developed PSE. Important components such as how the individual chooses the profession, the status of that profession, the society’s view of the profession, working conditions, the sufficiency of income, how authority/responsibility, and autonomy are used, and job security affect the PSE levels [6].

In Turkey, over two million students take nationwide Higher Education Institution Examinations annually to be admitted to universities after completing their high school education. After the examination, a fraction of the students can enroll in the department and university of their choice according to their scores on these examinations [7]. Most students study in programs that they do not target or desire for reasons such as having a university degree or increasing their job opportunities. Studies conducted in various provinces of Turkey reveal that the scores obtained from the university entrance examination, families’ wishes, and job opportunities are significant factors in choosing the departments of midwifery [8,9]. Midwives act in parallel with their PSE while providing care and service to women, fetuses, and newborns, applying all protective and preventive interventions, evaluating the results, and providing relevant guidance when deemed necessary [8,9,10]. Considering their reasons for choosing the profession, intense working conditions in the field, clinical settings, and academia, problems related to employment, complications related to their legal authority and responsibilities, role conflicts and uncertainties, and excessive workload, it is thought that midwives’ PSE may be adversely affected [8,9,10].

This study aims to determine the perception of PSE among midwifery students, explain how their perceptions are formed, and reveal how they perceive the midwifery profession and how the components that make up this perception determine their PSE. The following research questions were addressed: (1) What is the level of PSE among midwifery students? (2) How do students perceive the midwifery profession?

## 2. Methods

This study adopted or used an explanatory sequential design, which is a mixed-method design. The first stage of the study was conducted in a cross-sectional manner to determine the PSE of students. The second phase was conducted using an interpretive design, one of the qualitative research designs, about how students perceive the midwifery profession and how the components that make up this perception determine their PSE. The research design was to reveal what components affect the students’ PSE and what kind of perspective these components created in these students. In the first stage of the research, quantitative data were collected and analyzed. In the second stage, students’ perceptions of the midwifery profession were revealed with qualitative data, which was intended to help explain the quantitative data.

### 2.1. Population

The population of the study consisted of a total of 414 students: 113 in the first year, 101 in the second year, 104 in the third year, and 96 in the fourth year, who were studying in the fall semester at the largest state university in western Turkey in the 2020–2021 academic year. In the study, it was aimed to reach the entire universe, of which the sample was not selected. The criteria for inclusion in the study are being a student at Ege University’s Faculty of Health Sciences Department of Midwifery, being a volunteer to participate in the research, being in the group with the highest total score on the PSES (for the qualitative phase of the research), being in the group with the lowest total score on the PSES (for the qualitative phase of the research), and being willing to be interviewed (for the qualitative phase of the research). The exclusion criteria from the study were refusal to participate in the study, the students having sight, hearing, physical disability, or any psychiatric diagnosis, and being unsuitable for the interview. The quantitative part of the study included a total of 368 students enrolled in the undergraduate midwifery program. The qualitative part of the study was conducted with 17 students in total, including at least three students from each of the first, second, third, and fourth-year students with the highest and lowest total scores on the Professional Self-Esteem Scale (PSES).

### 2.2. Data Collection

The quantitative and qualitative data collection was entirely conducted face-to-face. In the first stage of the study, the ‘Individual Data Collection Form’ and the PSES were applied to midwifery students who agreed to participate in the study. While collecting data, it was explained to the students that they would be interviewed with a ‘Semi-Structured Interview Form’ and they were asked to write their nicknames on the data collection forms voluntarily. The total PSES score was calculated from the quantitative data obtained in the first stage. In the second stage, a total of 24 students were selected, including the three students with the highest and lowest total PSES score in each class level. From these, 17 students agreed to be interviewed via the Semi-Structured Interview Form. Interviews were recorded with the sound recording feature of a Samsung A50 mobile phone in the faculty meeting room. The transcriptions of the recorded qualitative data were prepared. The average number of pages of the interview texts was 6.47 ± 1.17 (min = 5, max = 9).

Individual Data Collection Form: This form consists of 12 questions to determine the socio-demographic characteristics of the students (seven questions) and the characteristics that are considered to affect their PSE (five questions).

Professional Self-Esteem Scale (PSES): The PSES was developed by Aricak (1999) and consists of 30 items that measure the respect and attitudes toward a profession among individuals aged 17 and over who have chosen that profession and are being trained in the respective area or are practicing that profession. Of these five-point Likert type items, 14 are positive statements (2, 5, 7, 9, 11, 13, 14, 16, 18, 20, 24, 26, 28) and 16 are negative statements (1, 3, 4, 6, 8, 10, 12, 15, 17, 19, 21, 22, 23, 25, 27, 29). Responses to the positive items are scored as 5 (Totally agree), 4 (Agree), 3 (Undecided), 2 (Disagree), and 1 (Strongly disagree) points. Responses to the negative items are scored as 1 (Totally agree), 2 (Agree), 3 (Undecided), 4 (Disagree), and 5 (Strongly disagree) points. The total score is obtained by adding the points given to each item; the lowest total score is 30 and the highest total score is 150. The PSE increases with the total score. The Cronbach’s alpha reliability coefficient of the scale was stated as 0.93 [2].

Semi-Structured Interview Form: This form included four open-ended questions to determine how students perceive the midwifery profession and what the components are that make up their perceptions: Opinions about the midwifery profession, the process of deciding to choose the midwifery undergraduate program, opinions about midwifery education, and the perception of Turkish society regarding the midwifery profession.

### 2.3. Data Analysis

The quantitative data was analyzed using SPSS Statistics for Windows, Version 25.0. The descriptive characteristics of the students are given with percentage distributions. One-way Analysis of Variance (ANOVA) was used to find out the difference between the overall scale score and the selected variables (class level, education of parents, type of high school graduated from, and reason for choosing the midwifery department) of the participants. An independent samples *t*-test was used to find the difference in the total PSES score of the participants and whether they had a relative who was a healthcare professional, chose the midwifery department willingly, and were convinced that they could perform the midwifery profession (before/after starting the midwifery department). Chi-square analysis was used to compare participants’ confidence in their ability to perform the midwifery profession in their own statements before and after starting the midwifery department. A *p*-value lower than 0.05 was sought for statistical significance.

Individual in-depth interviews were conducted through a semi-structured interview form using the face-to-face interview method. The qualitative data recorded in the audio were transcribed by the researchers. The data were analyzed in the MaxQda 2020 qualitative data analysis program by researchers with experience in using the program. In qualitative data analysis, the coding paradigm used by grounded theory and the coding paradigm of Straus and Corbin were used [11]. Open coding (open code) and categorization (exen code) were made and then themes (selective code) were created. In the creation of the themes, a common consensus of the researchers was achieved, and the theme names were created in accordance with the literature. After the themes were created, expert opinion was taken. The themes were finalized according to the suggestions of the experts. The interviewed students were identified with the letter “I”, those with low PSES scores were indicated with “L”, those with high PSES scores were indicated with “H”, and the order of interview was numbered as 1, 2, 3, …, and 17. For example, the participant coded as IL1 is the first interviewed student with the lowest PSES score.

## 3. Results

### 3.1. Demographic Results

The mean age of the 368 students who participated in the quantitative part of the study was 20.86 ± 2.07 years (range: 18–34 years). Of these, 87.5% had a nuclear family structure, and 84.5% had a medium family income level. The mothers of 46.5% of the students and the fathers of 32.3% were primary school graduates. Students’ socio-demographic characteristics are presented in Table 1.

The proportion of those who willingly chose the midwifery department was 80.4%, and 56.8% of these students consciously and enthusiastically chose the midwifery department. The rate of believing that they can perform the midwifery profession was 50.5% before they started their midwifery education, but it was 90.2% after they started the training. Students’ academic characteristics are presented in Table 2.

### 3.2. Quantitative Results: Students’ Professional Self-Esteem

The mean PSES score was 117.51 ± 17.60, indicating that students had a high PSE. The T-test analysis result shows that there was a significant difference between the PSES score and choosing the midwifery department willingly, believing that they could perform the midwifery profession before and after starting the midwifery education. According to the variance analysis, there was a significant difference between the PSES score and the reason for choosing the midwifery department (Table 3).

### 3.3. Qualitative Results

The thematic analysis carried out in the second phase of the study enabled a clearer interpretation of the components that may affect midwifery students’ PSE. In the first stage of the study, the difference between the reasons for choosing the midwifery department was found to be statistically significant. In the interviews conducted in the second stage, it was found that all of the students had to choose the midwifery department because their university entrance examination scores were not sufficient to get into their first choice and the scores required for the midwifery department were lower. Most of the interviewed students (n = 11) stated that their families had a significant impact on their choice of midwifery department. Families wanted their children to study in the midwifery department due to better opportunities to find a job after graduation. All of the interviewed midwifery students stated that the midwifery profession is perceived as a simple and low-status profession in Turkey; it is seen as a nursing profession; it is hierarchically positioned below physicians; and the profession is portrayed with a negative image in the media. Students also stated that midwives have important problems, such as receiving low salaries and not being independent in their responsibilities and exercising their powers. However, the students stated that they were happy to be in the midwifery department. According to the qualitative data, all of the students considered midwifery a sacred and professional career (n = 17) and were confident that they could perform the midwifery profession. Three main themes were obtained: (1) the meaning of being a midwife, (2) deciding to become a midwife, and (3) the difficulties of being a midwife.

Theme 1: Meaning of being a midwife

Students believed that midwifery was a sacred profession. They stated that they learned more about the profession during their education, that midwifery provides professional care to the woman giving birth and thus is an important profession, and that they were pleased to study in the midwifery department. Some of the statements were as follows:

*“We (midwives) play a direct role at the beginning of life. Midwifery is a very sacred profession. You help a living thing to come into this world, so I think midwifery is a very sacred profession.” *

[IH16]

*“Nurses provide care. We (midwives) support the mother and the baby, we help them give birth. While the nurses are dealing with a single life, we (midwives) have two lives to take care of and work on. Therefore, midwifery is a sacred profession for me. People around me think that nurses and midwives do the same job. I tell them that I will deal with the baby, the pregnant woman, the mother… I am happy to study in the midwifery department. Because now I am more aware, I know what kind of profession midwifery is. You are raising us very well equipped… I can give breastfeeding advice to a mother, I know what a midwife can do… I know much more than I did during the first-year internships.” *

[IL7]

Theme 2: Deciding to become a midwife

While the students explained how they chose the midwifery profession, they actually expressed the underlying conditions that drove them to midwifery. The majority of students had middle-income families. Families in Turkey care about their children having a job and expect support from their children when they have any health problems. Specifically, girls contributing to the household economy and housework and having a healthcare-related profession is regarded as a guarantee for their parents at an older age. Families encouraged their children to have a midwifery education since they thought that their daughters, if they scored low on the university entrance examination, would not only get a university education but also start working in a healthcare institution right after graduation.

*“There are more opportunities to find a job in healthcare professions such as nursing and midwifery compared to other professions. That’s why I chose (midwifery department).” *

[IL9]

*“I chose the midwifery department at the request of my mother and older sister. Actually, I wanted to study to become something like police or military officer, but I had problems with my eyes. That’s why my family wanted me to study in a health-related department at that time. We thought that I wouldn’t be unemployed when I graduated from university. Even if I can’t be assigned to public hospitals, we can work in private hospitals.” *

[IH10]

Theme 3: Difficulties of being a midwife

By emphasizing the problems and social image of the midwifery profession, the students clearly expressed the difficulties they faced after choosing the midwifery department. They frequently stated that midwifery is perceived as a simple profession in society and, therefore, a profession with a low social status. The negative perception of the society about the midwifery profession is expressed as being positioned at lower levels in the hierarchy of medical professions, with barriers preventing midwives from taking on responsibilities and being independent in exercising their powers, as well as very low salaries. The majority of the students stated that the responsibilities of the midwifery and nursing professions are not clear and that the midwifery profession is the same as the nursing profession in the view of society. The students stated that midwives are not promoted with a good image in the media and that it is not known by society that a university education is required to become a midwife. In addition, the participants indicated that there are regulatory problems such as the lack of legislation for the midwifery profession, employment, and challenging work conditions.

*“People don’t know the value of midwives. They say, “Is there still a midwifery (position)?” They despise it. They are surprised when they hear that I studied midwifery at university.” *

[IH4]

*“I think that midwives are undervalued in Turkey. The problems of the midwifery profession are related to management. Doctors do the work that midwives should do and they suppress midwives.” *

[IH12]

## 4. Discussion

This study investigated the PSE of the students at the midwifery department in the largest state university located in western Turkey, how they perceive the midwifery profession, and how the components of this perception shape their PSE. At the quantitative stage of the study, the students’ mean PSES scores were measured and found to be high. In the qualitative stage, interviews were conducted with students with the lowest or highest PSES scores, aiming to reveal how the differences in these students’ perceptions of the midwifery profession emerge. However, regardless of their PSES scores, all interviewed students used similar expressions and emphasized common positions and problems. In general, both quantitative measurements and qualitative analyses revealed that students’ PSE was high despite the existence of many problems related to the midwifery profession in Turkey.

Being one of the oldest professions, the midwifery profession has undoubtedly gone through significant changes and transformations. These changes produced today’s occupational conditions, reinforcing midwifery as a scientific and professional occupation, as well as the fact that it is actually a powerful, respectable, and human-oriented profession. This reality has enabled midwifery to be identified as a sacred profession [12,13]. Beyond helping her give birth, midwifery is witnessing the rebirth of a woman. This witnessing enables the midwife to love human beings, reduce the birthing process to the laws of nature, and care about emotional and spiritual balance [14]. During their training, midwifery students get to know their profession better, decide whether the profession is suitable for them, and form an emotional bond with the profession [15]. In a quantitative study in Turkey, it has been stated that midwifery students mostly like the profession, have a positive view of the profession, find the profession suitable for them, are pleased to study in this field due to its “helping people” aspect, and therefore, the PSE is high among those who chose the profession [16]. In this study, the interviews demonstrated that the students liked the midwifery profession and were pleased to be able to carry out the profession. Students think that midwifery is a profession that serves women, fetuses, and newborns in particular, and society in general. They think that society’s awareness of the importance of midwifery services and its demand for these services should increase. They emphasized that midwives should not only share accurate and reliable information in the social information programs but also convey this information during the services they provide. At the same time, they believe in the sanctity of being able to help and support women during childbirth, a time of special meaning for them. All these components are thought to increase students’ PSE.

In Turkey, high-school graduates specify the vocational programs and universities they can enter by making twenty-four choices. They gain the right to enroll in a department based on their score on the university entrance examination and their choices. According to the data from the Council of Higher Education for the year 2021, the order of preference for midwifery programs ranges from eight to twenty-four for the students studying in the midwifery departments [17]. Thus, it can be said that most of the students enrolled in the midwifery programs do not prefer to study in that program as their primary choice. A conscious career choice enables one to complete vocational education with a solid foundation, fulfill the requirements of the profession, and have a high PSE [18]. In the first stage of this study, four-fifths of the students stated that they chose the midwifery department voluntarily, and more than half of them stated that they chose the midwifery profession consciously and in line with their wishes (56.8%). However, in the interviews conducted in the second stage of the study, it was revealed that the students chose the midwifery profession as a result of the requests or guidance of their families. The most important factor in deciding to study in the midwifery department was getting a low score on the university entrance examination and better job opportunities after graduation. Although the participants stated that studying in the midwifery program was their choice, it can be said that the parents’ concerns about their children being unemployed significantly affected their decision. Studies conducted in different cities in Turkey showed that students mostly preferred the midwifery department because of their families’ wishes and the opportunity to work immediately after graduation [8,10,13,19]. Studies indicated that the midwifery students pick the department as their last choice or reluctantly. It was found that 43.1% of the students studying midwifery at a university in the Central Anatolian region of Turkey chose the department voluntarily, but none of them had it as their first choice; another study in the same region found that 43% of the students listed the midwifery department as their last pick [10,19]. In another study conducted by reaching students from all midwifery departments in Turkey, it was found that most of the students were in the midwifery department reluctantly [20]. It is thought that families in Turkey have become more concerned about finding a job right after graduation due to the rapid increase in the number of universities and graduates in recent years and low employment rate for educated individuals. In addition, factors such as the higher rate of employment as a public servant through appointments made every two years at regular intervals, the increase in the number of private hospitals and the possibility of starting work in these hospitals immediately after graduation—albeit at lower wages—the common belief in Turkish society that having a family member who is healthcare personnel will benefit the family, and the fact that midwifery has a more defined set of responsibilities, such as maternal and child health services, are thought to be effective in the families’ inclination to encourage students to pursue the midwifery profession.

The most frequently cited problem in the interviews was the low social status of the midwifery profession. The students stated that they often heard negative statements about the midwifery profession from their social environment. They stated that, when they talk about being a midwifery student, the people in their social environment indicate that it is the same as nursing, midwives receive low salaries, it is a high school education, and they are positioned as a profession below the status of doctors and nurses. The expectations and perspectives of society play a decisive role in the development and delivery of high-quality healthcare services [21]. The main reasons for the low status of the midwifery profession around the world are gender inequality and insufficient economic and political investments in education, practice, regulation, and licensing processes [22,23,24]. A study conducted in Ethiopia found that students did not choose the midwifery profession because the status of the profession was low [25]. It has been stated that midwifery students in India continue their education under the influence and authority of the medical profession [26]. Midwifery students in the Mediterranean region of Turkey stated that society did not know about the midwifery profession or the duration of midwifery training [27]. Studies conducted in different regions of Turkey presented similar results [21,27,28]. One of the major challenges for midwifery students is their efforts to continue learning in a society dominated by physicians, even though they will be serving as partners within the same healthcare system. This dominance causes midwife students to have limited opportunities for practical applications during their training. Midwives think that they will be positioned at a lower level in the existing medical hierarchy in the healthcare sector after students graduate. This hierarchical structure can be generally attributed to gender inequality and the treatment and physician-centered delivery of healthcare services [22,26]. In Turkey, midwifery education was given at the secondary-school level in the 1960s, high school level in the 1970s, and at the two-year associate degree level in the 1980s. Since 1997, midwifery training has involved a four-year university education [29]. As it has been offered only for the last twenty-five years, midwifery is relatively new as a university education compared with other healthcare professions, and social awareness may not have developed. However, it is evident that there are important deficiencies in the promotion of the profession on a social level. In addition, it is anticipated that the social perception of the profession will evolve in a positive direction with the provision of more qualified midwifery services by midwives who are trained as professional members of the healthcare workforce.

The expressions used by all of the students show that they believe that solutions can be found for the problems of the midwifery profession and that the status of the midwifery profession in Turkey can be improved. The participants thought that the midwifery education they receive and the qualified midwifery services they will provide after graduation will increase the status of the profession in the future. It was thought that this optimism affected their PSE positively. Other studies have also shown that students had positive opinions about the midwifery profession throughout their education, considered it a suitable profession for them, and were happy about choosing the midwifery department [16,27]. It can be said that midwifery education allows students to increase their awareness and recognize the profession as a whole in theory and practice, and at the same time, it contributes to their PSE by giving students professional awareness.

## 5. Conclusions

This study determined the level of professional self-esteem among midwifery students and revealed how it was shaped. Despite the existence of several problems related to the midwifery profession in Turkey, it has been revealed that the professional self-esteem of the students is high. Choosing a profession willingly and believing that one can do the profession are important factors that increase professional self-esteem.

High professional self-esteem benefits midwifery students will increase the quality of care given to women, fetuses, and newborns in every area where midwives are involved in the clinic and academia. Students think that the midwifery profession is important and has a high spiritual aspect. However, the participants think that there is not enough awareness in the society about the midwifery profession in Turkey and that there are many difficulties facing the profession. Raising awareness about the importance of the midwifery profession in the general population and creating a midwifery directorate within the ministries of health can improve professional self-esteem. Health politicians, educators, academics, and clinicians can benefit from this work.

## 6. Strengths and Limitations

The mixed-methods design of this study helped to explore not only the average PSE of students’ midwives but also the contributory themes. By combining both qualitative and quantitative methodology, we were able to offer a more comprehensive picture of PSE among student midwives than we could have with either method alone. However, this mixed-methods study is not without limitations. This study only reflects the views of student midwives, and midwive’s perspectives were not included. Similar studies could be conducted among midwives to compare the results.

## Figures and Tables

**Table 1:** Students’ demographic characteristics (n = 368).

Characteristics	n	%
Age		
15–19	81	22.0
20–24	272	73.9
25–29	11	3.0
30–34	4	1.1
Family Type		
Nuclear family	322	87.5
Extended family	46	12.5
Family Income Level		
Below medium	18	4.9
Medium	311	84.5
Above medium	39	10.6
Educational of Mother		
Primary school graduate or lower	223	60.6
Secondary school graduate	126	34.2
High school graduate or above	19	5.2
Educational of Father		
Primary school graduate or lower	144	39.1
Secondary school graduate	174	47.3
High school graduate or above	50	13.6
Type of High School Graduated		
High school	71	19.3
Anatolian/Science high school	260	70.7
Health vocational high school	37	10.1
Class Level in the Midwifery Department		
First-year	93	25.3
Second-year	94	25.5
Third-year	89	24.2
Fourth-year	92	25.0
Total	368	100

**Table 2:** Students’ academic characteristics (n = 368).

Characteristics	n	%
Choosing the Midwifery Department Willingly		
Yes	296	80.4
No	72	19.6
Reason for Choosing the Midwifery Department		
My conscious choice	209	56.8
Job security	95	25.8
My family’s request	41	11.1
Other	23	6.2
Believing that they can perform the midwifery profession (before starting the midwifery education)		
Yes	186	50.5
No	182	49.5
Believing that they can perform the midwifery profession (after starting the midwifery education)		
Yes	332	90.2
No	36	9.8
Total	368	100

**Table 3:** Comparison of the mean PSES scores and variables.

Variables	PSES Score	t/F	*p*
	M ± SD		
Age			
15–19	119.19 ± 17.12	0.534	0.710
20–24	116.86 ± 17.74		
25–29	119.63 ± 20.05		
30–34	117.66 ± 11.23		
Class Level in the Midwifery Department		2.391	0.068
First-year	117.38 ± 17.08		
Second-year	117.43 ± 18.34		
Third-year	119.98 ± 14.83		
Fourth-year	115.32 ± 19.66		
Type of High School Graduated		0.858	0.425
High school	115.49 ± 17.83		
Anatolian/Science high school	118.26 ± 16.78		
Health vocational high school	115.97 ± 22.34		
Choosing the Midwifery Department Willingly		11.134 *	0.000
Yes	121.87 ± 15.52		
No	99.58 ± 13.95		
Reason for Choosing the Midwifery Department		43.041	0.000
My conscious choice	125.14 ± 15.18		
Job security	104.21 ± 17.02		
My family’s request	110.07 ± 14.02		
Other	102.60 ± 16.34		
Believing that they can perform the midwifery profession (before starting the midwifery education)			
Yes	122.91 ± 17.04	6.257 *	0.000
No	111.98 ± 16.45		
Believing that they can perform the midwifery profession (after starting the midwifery education)			
Yes	119,87 ± 16,36	8.554 *	0.000
No	95,72 ± 13,29		

* independent *t*-test.

## Data Availability

The datasets used and analyzed during the current study are available from the corresponding author on reasonable request.
